# 484. Clinical Description of SARS-CoV-2 Infection in Patients with Primary Antibody Deficiency Who Received Tixagevimab/Cilgavimab Prophylaxis

**DOI:** 10.1093/ofid/ofad500.554

**Published:** 2023-11-27

**Authors:** Sung Min Lim, Je Hee Shin, Jee Yeon Baek, Ji Young Lee, Ji-Man Kang, Jong Gyun Ahn

**Affiliations:** Hanyang University Hospital, Hanyang University College of Medicine, Gimpo, Kyonggi-do, Republic of Korea; Severance Children’s Hospital, Yonsei University College of Medicine, Seoul, Seoul-t'ukpyolsi, Republic of Korea; Yonsei University College of Medicine, Seoul, Seoul-t'ukpyolsi, Republic of Korea; Yonsei University College of Medicine, Seoul, Seoul-t'ukpyolsi, Republic of Korea; Severance Children’s Hospital, Yonsei University College of Medicine, Seoul, Seoul-t'ukpyolsi, Republic of Korea; Severance Children’s Hospital, Yonsei University College of Medicine, Seoul, Seoul-t'ukpyolsi, Republic of Korea

## Abstract

**Background:**

Patients with primary antibody deficiency are known to be associated with a higher risk of SARS-CoV-2 infection, complications, and mortality. Tixagevimab/Cilgavimab prophylaxis has shown efficacy in reducing hospitalization and mortality. We aim to describe the clinical characteristics and outcomes of COVID-19 cases in primary antibody deficiency patients receiving tixagevimab/cilgavimab prophylaxis in South Korea.

**Methods:**

This is a descriptive analysis of COVID-19 infection in primary antibody deficiency patients at Severance Children's Hospital. From September 2021 to February 2023, we retrospectively collected data, including demographic characteristics, comorbidities, tixagevimab/cilgavimab prophylaxis status, SARS-CoV-2 vaccination, COVID-19 infection, clinical presentation, COVID-19 treatment, oxygen supplement, hospitalization, and clinical outcome.

**Results:**

Thirteen patients (12 with X-linked agammaglobulinemia and 1 with common variable immunodeficiency) were enrolled in this study. Among them, 10 received tixagevimab/cilgavimab prophylaxis, while the remaining 3 did not. Three patients developed breakthrough infection after receiving prophylaxis. Patient 1 (29 years old, male) completed a SARS-CoV-2 booster mRNA vaccination, had no prior COVID-19 infection, and developed COVID-19 infection 104 days after prophylaxis. Patient 2 (33 years old, male) received 2 doses of mRNA vaccine, had no prior infection history, and developed COVID-19 infection 3 days after prophylaxis. Patient 3 (13 years old, male) had no history of previous vaccination, had an infection history before receiving prophylaxis, and developed COVID-19 infection 19 days after prophylaxis. All 3 patients experienced mild symptoms and did not require oxygen therapy or hospitalization. Of the 7 patients who were not infected with COVID-19 after prophylaxis, 6 had a history of COVID-19 infection, and all of them received SARS-CoV-2 mRNA vaccine. The follow-up period after prophylaxis ranged from 198 to 241 days.

**Table 1.**
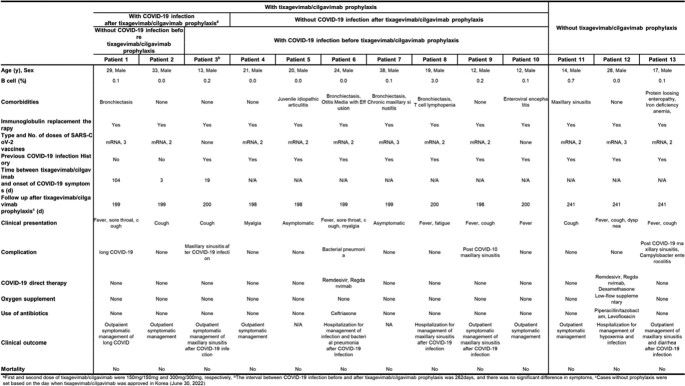
Clinical description of the hypogammaglobulinemia patients who were diagnosed with COVID-19 with or without tixagevimab-cilgavimab prophylaxis

**Conclusion:**

In this study, we observed that 3 out of 10 primary antibody deficiency patients who received tixagevimab/cilgavimab prophylaxis were infected COVID-19 but did not require hospitalization or oxygen supplementation.

**Disclosures:**

**All Authors**: No reported disclosures

